# *GNG7* and *ADCY1* as diagnostic and prognostic biomarkers for pancreatic adenocarcinoma through bioinformatic-based analyses

**DOI:** 10.1038/s41598-021-99544-x

**Published:** 2021-10-14

**Authors:** Youfu Zhang, Jinran Yang, Xuyang Wang, Xinchang Li

**Affiliations:** Department of Organ Transplantation, Jiangxi Provincial People’s Hospital Affiliated To Nanchang University, No. 92 The Aiguo Road, Nanchang, 330006 Jiangxi Province People’s Republic of China

**Keywords:** Cancer, Genetics

## Abstract

Pancreatic adenocarcinoma (PAAD) is one of the most lethal malignant tumors in the world. The GSE55643 and GSE15471 microarray datasets were downloaded to screen the diagnostic and prognostic biomarkers for PAAD. 143 downregulated genes and 118 upregulated genes were obtained. Next, we performed gene ontology (GO) and The Kyoto Encyclopedia of Genes and Genomes (KEGG) analysis on these genes and constructed a protein–protein interaction (PPI) network. We screened out two important clusters of genes, including 13 upregulated and 5 downregulated genes. After the survival analysis, 3 downregulated genes and 10 upregulated genes were identified as the selected key genes. The KEGG analysis on 13 selected genes showed that *GNG7* and *ADCY1* enriched in the Pathway in Cancer. Next, the diagnostic and prognostic value of *GNG7* and *ADCY1* was investigated using independent cohort of the Cancer Genome Atlas (TCGA), GSE84129 and GSE62452. We observed that the expression of the *GNG7* and *ADCY1* was decreased in PAAD. The diagnostic receiver operating characteristic (ROC) analysis indicated that the *GNG7* and *ADCY1* could serve as sensitive diagnostic markers in PAAD. Survival analysis suggested that expression of *GNG7*, *ADCY1* were significantly associated with PAAD overall survival (OS). The multivariate cox regression analysis showed that the expression of *GNG7*, *ADCY1* were independent risk factors for PAAD OS. Our study indicated *GNG7* and *ADCY1* may be potential diagnostic and prognostic biomarkers in patients with PAAD.

## Introduction

Pancreatic adenocarcinoma (PAAD) is one of the most malignant cancers. It was reported that PAAD accounts for more than 0.33 million deaths per year^1^. The 5-year survival rate of PAAD patients is still less than 5%, and the survival time of patients after multi-drug chemotherapy is generally still less than 1 year^2^. The cause of pancreatic adenocarcinoma is currently unknown. Studies have found that the occurrence of chronic pancreatitis is one of an important risk factor for PAAD^3,4^. Previous studies have also found that the occurrence of PAAD is obviously related to the intestinal flora, especially the types of oral microorganisms^5,6^. Early diagnosis and treatment of pancreatic cancer are of great importance to prolong the survival time of patients.

By reason of the lack of sensitive early diagnosis methods, it is challenging to develop effective treatments to delay and reverse the progression of PAAD. Serum CA19-9 is of great value for the diagnosis of PAAD and for assessing tumor progression, but it is not recommended for general screening because it is elevated in non-tumor diseases such as chronic pancreatitis and acute cholangitis^7,8^. However, CA19-9 is still considered to be the most helpful tumor marker for the diagnosis and prognosis for PAAD. In recent years, plenty of researches was devoted to identifying the diagnostic and prognostic biomarkers for PAAD. Lv et, al. reported that high *TXLNA* expression predicts favorable outcomes for PAAD patients^9^. Zheng and his colleagues identified *COL11A1* as an immune infiltrate correlated prognosticator in PAAD through integrated bioinformatics analysis^10^. Nevertheless, biomarkers for the diagnosis and prognosis for PAAD are still lacking due to its heterogeneity^11^.

This present study aimed to screen out the genes that has diagnostic and prognostic significance for PAAD. We downloaded the GSE55643 and GSE15471 mRNA expression profiles from the Gene Expression Omnibus (GEO) database and then used built-in packages such as limma^12^ (version 3.22.1; Fred Hutchinson Cancer Research Center) with R software^13^ to perform differentially expressed genes (DEGs) analysis between the primary tumor tissue of PAAD patients and the paired adjacent non-malignant tissue. Next, we used Venn plots to query the overlapping DEGs obtained from GSE55643 and GSE15471. Then, the Database for Annotation, Visualization and Integrated Discovery (DAVID) database was used to perform the gene ontology (GO) and the Kyoto Encyclopedia of Genes and Genomes (KEGG) enrichment analysis on the overlapping DEGs^14^. We constructed the protein–protein interaction (PPI) network of DEGs via the Search Tool for the Retrieval of Interacting Genes (STRING) database to identify the highly interconnected protein molecules and visualized by Cytoscape software^15–18^. Finally, we investigated the association between the selected gene expression with clinical outcomes using independent PAAD patient data obtained from the Cancer Genome Atlas (TCGA) database, GSE84129, and GSE62452 datasets^19,20^. Through integrated bioinformatics approaches, we screened out two potentially valuable biomarkers for the diagnosis and prognosis of PAAD.

## Methods

### Acquisition and processing of microarray data

mRNA expression data from microarray files GSE55643^21^ and GSE15471^22^ were downloaded from the GEO database^23,24^. GSE55643 contains 45 primary and 8 adjacent non-malignant tissue samples from PAAD patients. GSE15471 contains 78 pairs of primary and para-cancerous tissue samples from PAAD patients. We downloaded the normalized log2 ratio representing tumor/normal of the GSE55643 and GSE15471 dataset and converted the probe identification numbers to gene symbols using the Whole Human Genome Microarray 4 · 44 K G4112F (Probe Name Version) and the Affymetrix Human Genome U133 Plus 2.0 Array, respectively.

### Identification of DEGs

We utilized the R software and built-in limma packages (version 3.22.1; Fred Hutchinson Cancer Research Center) to perform DEGs analysis between the primary tumor tissue of PAAD patients and the paired adjacent non-malignant tissue. DEGs must meet our two important criteria to be selected for the next stage of analysis: adjusted *P* value < 0.05, |log2 fold change (FC)|> 1.2^25^.

### KEGG and GO enrichment analysis of DEGs

The GO clarifies the functions of genes from three aspects: cellular component (CC), molecular function (MF), and biological process (BP)^26^. The KEGG provides data resources for understanding high-level functions and utilities of the biological metabolic pathways^27^. DAVID, a network server that provides a comprehensive set of functional annotation tools to determine the biological meaning of a large list of genes, was used for GO function annotation and KEGG pathway enrichment analysis^14^. *P* value < 0.05 was considered statistically significant.

### Construction of PPI networks and identification of gene clusters

STRING database (version11.0), containing more than 2 billion interactions of 24.6 million proteins involving more than 5,000 organisms, was used to search for DEGs’ encoded protein and PPI network information. We uploaded the overlapping DEGs from the microarray data GSE55643 and GSE15471 into the STRING database and the significance threshold was set to an interaction score of 0.900 (highest confidence). Then, we visualized the PPI network in Cytoscape software. Next, we used built-in Molecular Complex Detection (MCODE) of Cytoscape to screen out highly interconnected protein molecules as molecular clusters.

### Survival and expression analysis of gene clusters and KEGG enrichment analysis

The Kaplan Meier plotter is capable to assess the effect of 54 k genes (mRNA, miRNA, protein) on survival in 21 cancer types. Sources for the databases include GEO, EGA, and TCGA^19^. But for PAAD, the expression and survival data are all sources from the TCGA database (n = 177). The TNMplot database contains 56,938 unique multilevel quality-controlled samples of normal tissues, tumor tissues, and metastatic tissues from GEO, GTex/TCGA, and TARGET databases. We performed the Kaplan–Meier curve and log-rank test analyses to investigate the prognostic value of gene clusters in PAAD tissues. The TNMplot database (https://www.tnmplot.com/) was used for the comparison of gene expression in normal, tumor and metastatic tissues of PAAD patients. Then, the member of gene clusters validated by the Kaplan–Meier plotters and TNMplot database were screened as selected genes. Finally, the DAVID was used to perform the KEGG enrichment analysis on the selected genes to explore the pathway that may regulate the occurrence and development of PAAD.

### Diagnostic and prognostic significance of selected gene in TCGA and GEO database

We analyzed the expression of the selected genes in PAAD tissues and para carcinoma tissues from patients in the TCGA database and GEO database (GSE84129, and GSE62452 datasets). We performed the Kaplan–Meier curve and log-rank test and ROC analyses as well as multivariate Cox regression analysis to investigate the relationship between the expression of selected genes and the clinical outcome of PAAD patients.

### Statistical analysis

SPSS 21 (SPSS Inc., Chicago, IL, USA) was used to perform statistical analysis. GraphPad Prism 5.0 (GraphPad Software, Inc., San Diego, CA, USA) was used to draw figures. Pearson’s chi-square test was used to compare the categorical variables. The student’s t-test was used to analyze normally distributed continuous variables. Kaplan–Meier plots with the log-rank test were used to estimate survival differences. The diagnostic significance of selected genes for PAAD was evaluated by the ROC curve. *P* < 0.05 was considered statistically significant.

## Results

### Identification of DEGs in PAAD

**Figure 1:**
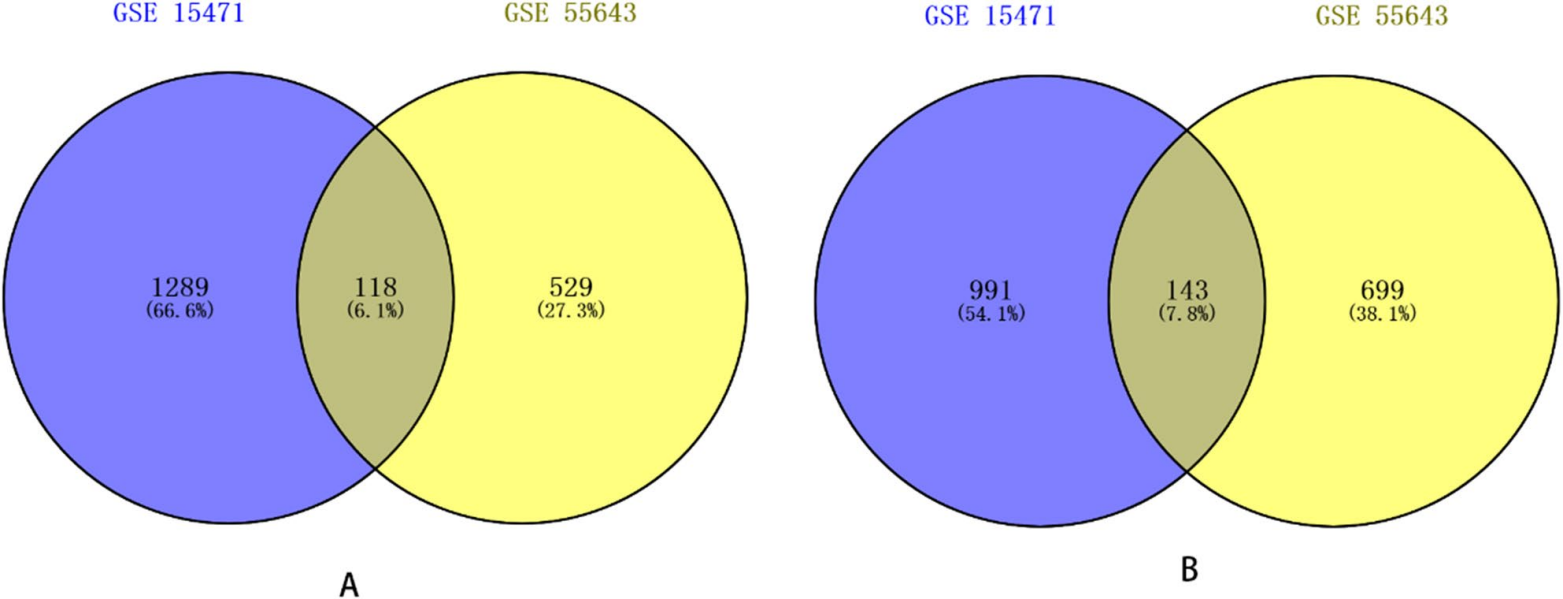
A Venn diagram showing the overlapping 261 DEGs in the two datasets (GSE15471 and GSE55643). (**a**) 118 up-regulated DEGs in the two datasets (log2FC > 1.2). (**b**) 143 down-regulated DEGs in two datasets (log2FC < -1.2).

We screened DEGs from 123 cases of primary tumor tissues using limma packages in R language, compared with 86 cases paired para-cancerous nonmalignant tissues. Using both adjusted *P *value < 0.05 and logFC (fold change) > 1.2 criteria, a total of 1228 genes were identified in GSE55643, including 529 up-regulated genes and 699 down-regulated genes. A total of 2280 genes were screened in GSE15471, including 1289 up-regulated genes and 991 down-regulated genes. Then, the overlapping DEGs in the microarray data GSE55643 and GSE15471 were analyzed by Venn plot (Fig. 1), and finally, 261 DEGs were obtained, including 118 up-regulated genes and 143 down-regulated genes (Supplementary Table 1).

### KEGG and GO enrichment analysis of DEGs

To further explore the function of the identified DEGs in PAAD, the online biological classification software DAVID was applied to perform GO and KEGG pathway analyses on PAAD. As shown in Supplementary Fig. 1, we list the top 6 significant terms for the BP, CC, MF, and KEGG pathways of DEGs, respectively (Supplementary Fig. 1). We list the annotation of the up-regulated genes and down-regulated genes. The up-regulated DEGs were mainly enriched for genes involved in the type I interferon signaling pathway, response to virus and extracellular matrix organization in the BP group. The down-regulated DEGs were mainly enriched genes in the reactive oxygen species metabolic process, regulation of cell adhesion and multicellular organism aging. In the CC group, the up-regulated DEGs were mainly enriched for genes associated with the extracellular exosome, extracellular space and extracellular region. The down-regulated DEGs were mainly enriched in the integral component of plasma membrane, extracellular exosome and integral component of membrane. In the MF group, the up-regulated DEGs were mainly enriched from genes associated with chemokine activity, laminin binding and calcium ion binding. The down-regulated DEGs were enriched in the lipid binding, phospholipid binding and serine-type endopeptidase inhibitor activity in the MF group. In addition, 3 significant KEGG pathways for up-regulated genes, such as ECM-receptor interaction, PI3K-Akt signaling pathway, focal adhesion. Moreover, 3 significant KEGG pathways for down-regulated genes, such as fat digestion and absorption, protein digestion and absorption, vitamin digestion and absorption (Supplementary Table 2).

### Construction of PPI networks and identification of gene clusters

**Figure 2:**
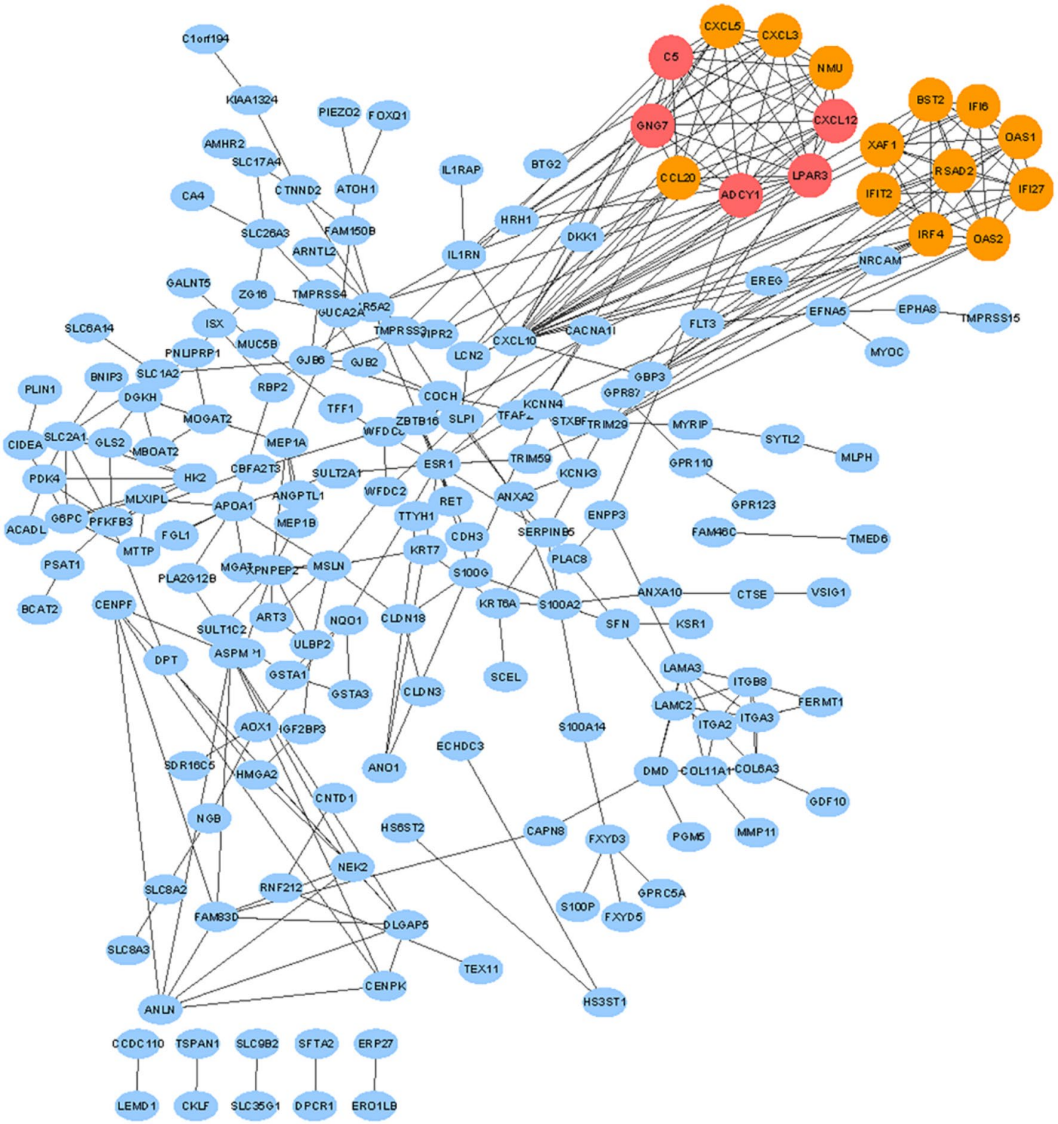
PPI networks of DEGs. Based on the STRING online database, 186 genes/nodes were filtered into the DEG PPI network and visualized by Cytoscape software (version 3.7.2). The two highlighted circle areas are the most significant modules. upregulated DEGs are represented by orange circles and downregulated DEGs are represented by red circles.

The 118 up‑ and 143 downregulated genes were input into the STRING database to identify the significant interactions between proteins and then visualized by Cytoscape software. 186 nodes with 348 edges were selected to construct the PPI networks with a confidence score of > 0.900 (highest confidence). 2 gene clusters both including 9 nodes with 36 edges were identified by MCODE which was built in the Cytoscape (Fig. 2). Gene cluster 1 includes five down-regulated genes (*C5, ADCY1, GNG7, CXCL12, LPAR3*) and four up-regulated genes (*CXCL3, CXCL5, CCL20, NMU*). All genes of gene cluster 2 were up-regulated genes (*IFIT2, IRF4, BST2, XAF1, OAS1, OAS2, IFI6, IFI27, RSAD2*).

### Survival and expression analysis of cluster of genes in the Kaplan–Meier plotters and the TNMplot database

**Figure 3:**
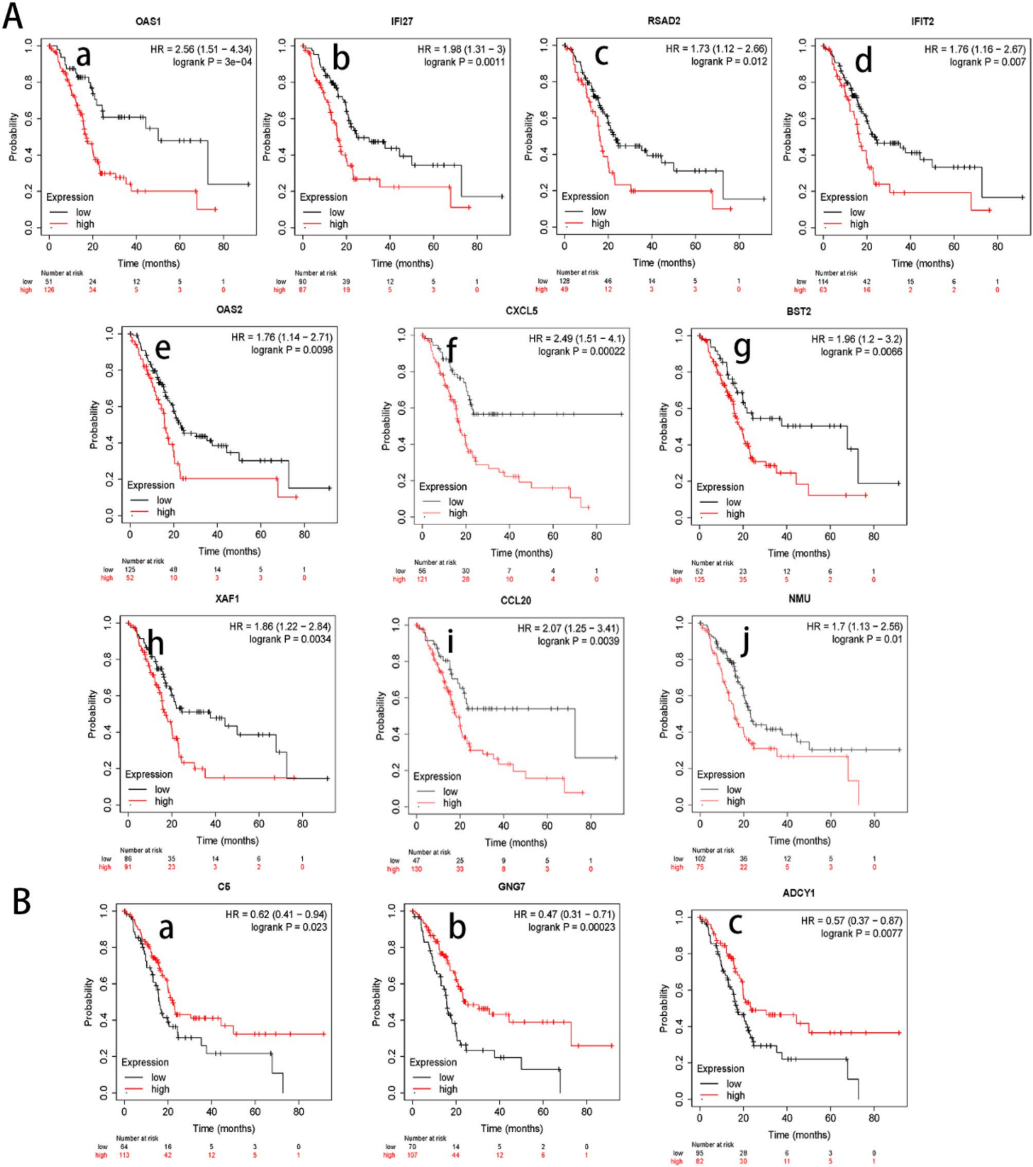
Overall survival of the genes with expression in accordance with that in Kaplan–Meier Plotter database (http://kmplot.com/analysis/). (**A**) High expression of 10 up-regulated genes was associated with poor overall survival in PAAD (a-j). (**B**) Low expression of 3 down-regulated genes was associated with poor overall expression in PAAD (a-c).

**Figure 4:**
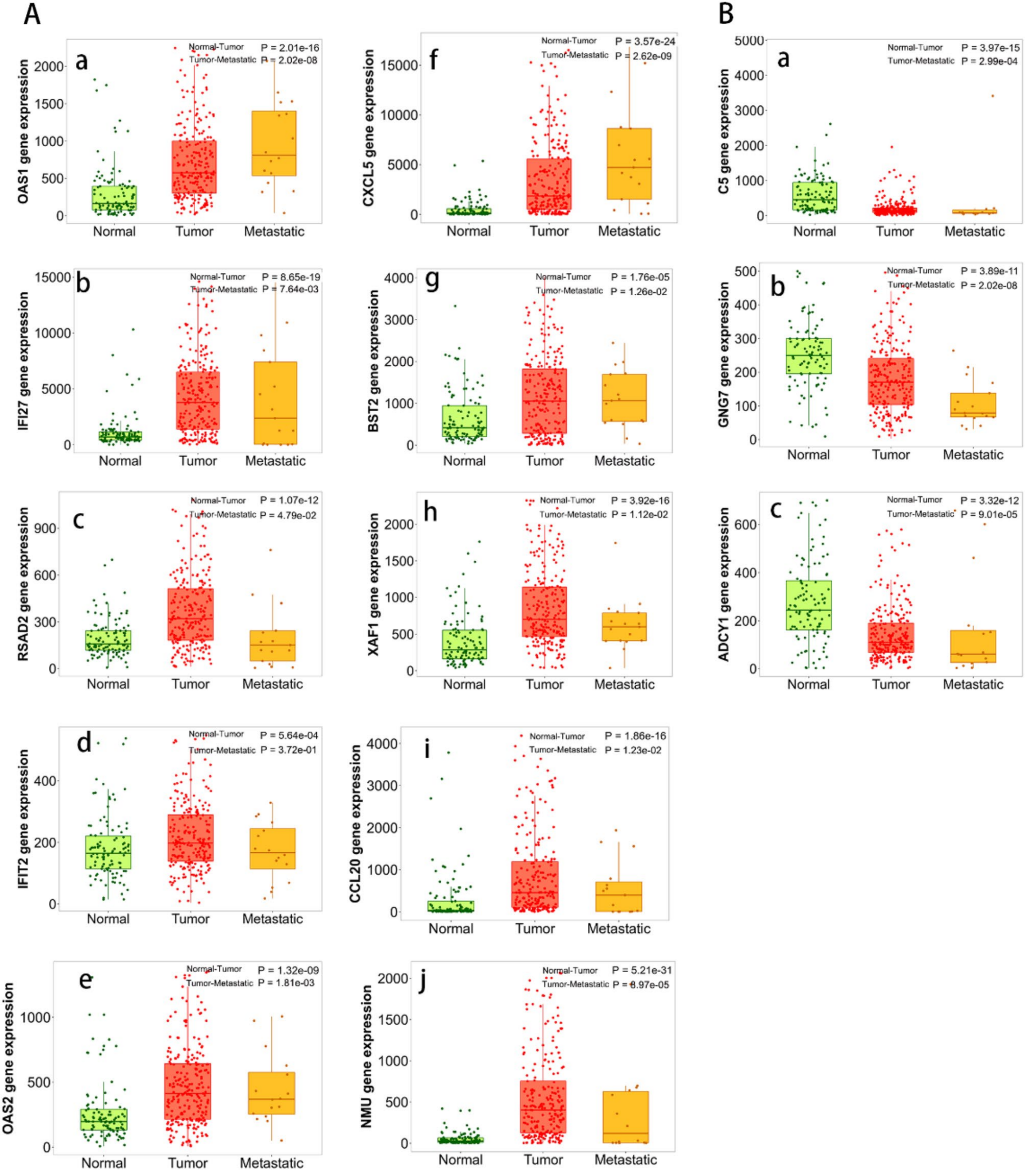
The comparison of the cluster of genes expression in normal, tumor, and metastatic tissues of PAAD patients in the TNMplot database (https://www.tnmplot.com/). The 177 PAAD specimens were divided into high expression group and low expression group taking the median expression as the critical point. (A) 10 up-regulated genes significantly overexpressed in PAAD samples compared with normal tissues (**a**–**j**). The mRNA expression of OAS1, CXCL5, and BST2 in metastatic tissues was higher than tumor tissues (**a**, **f**, **g**). (**B**)The mRNA expression of 3 down-regulated genes was incrementally downregulated in the normal, tumor, and metastatic tissues (**a**–**c**).

We performed the Kaplan–Meier curve and log-rank test analyses to investigate the prognosis of gene clusters in PAAD tissues. The 177 PAAD specimens were divided into high expression group and low expression group taking the median expression as the critical point. The results showed that 13 genes expression including 10 up-regulated genes (*XAF1, IFI27, OAS1, OAS2, IFIT2, RSAD2, BST2, CCL20, NMU, CXCL5,* Fig. 3A) and 3 down-regulated genes (*C5, ADCY1, GNG7*, Fig. 3B) were associated with significantly poorer overall survival in patients with PAAD, whereas 5 genes including 3 up-regulated genes (*IRF4, IFI6* and *CXCL3*) and 2 down-regulated genes (*CXCL12, LPAR3*) showed no significant correlations^19^. We also used the TNMplot database for the comparison of these genes expression validated by Kaplan–Meier curve and log-rank test analyses in normal, tumor, and metastatic tissues of PAAD patients. Compared with normal samples, 10 genes were significantly overexpressed (Fig. 4A) and 3 genes were significantly down expressed (Fig. 4B) in PAAD samples, which was consistent with the original datasets. In addition, *OAS1, CXCL5* and *BST2* expression levels were exhibited higher in PAAD metastatic samples than no metastatic samples. The down-regulated genes, *C5, GNG7*, and *ADCY1* were exhibited significant lower expression in metastatic samples than no metastatic samples.

### KEGG enrichment analysis of 13 selected Genes by DAVID.

**Figure 5:**
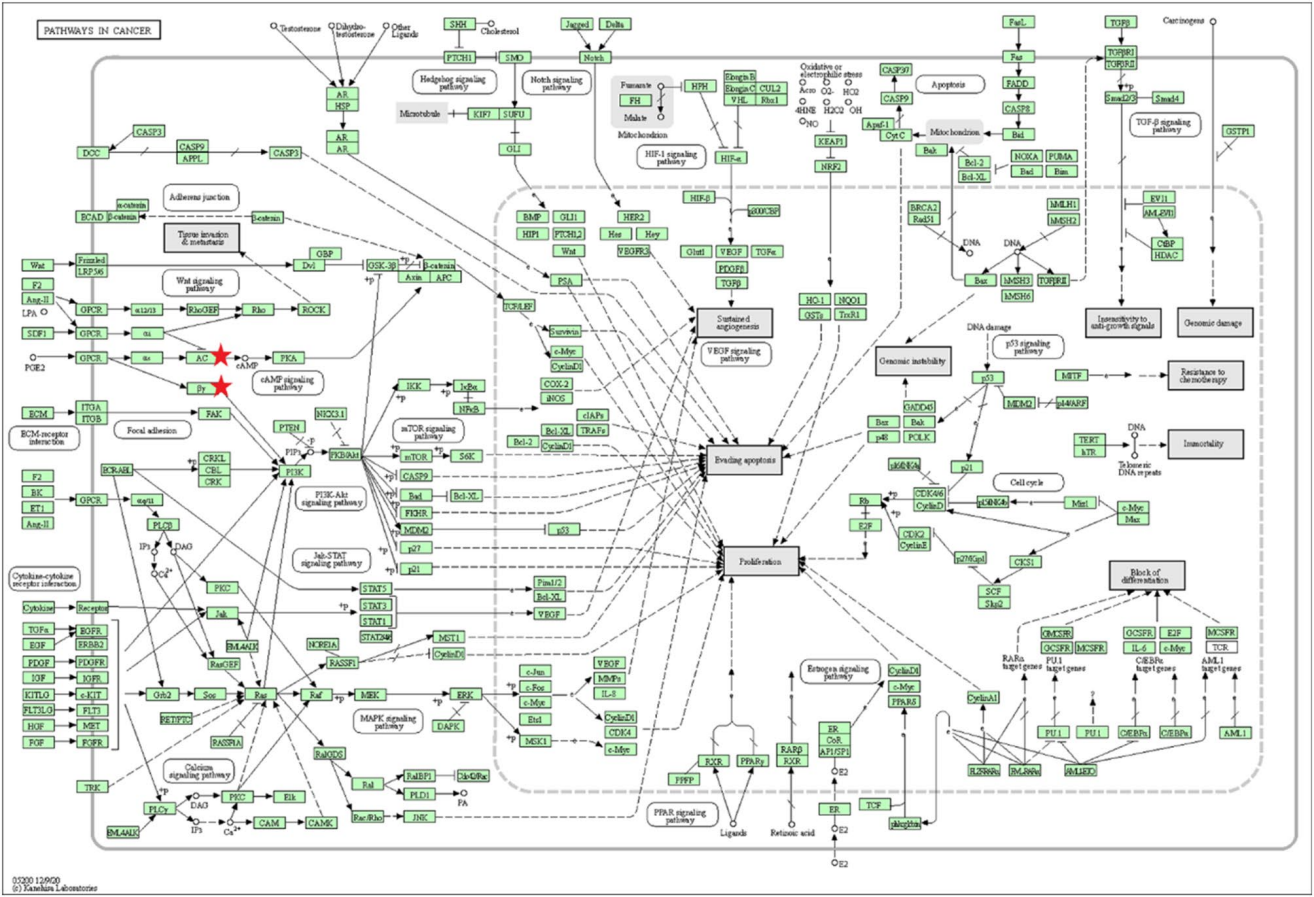
Reanalysis of 13 validated genes via KEGG pathway enrichment. two genes (*GNG7, ADCY1*) were markedly enriched in the Pathways in Cancer (https://www.kegg.jpkegg/kegg1.html). AC means *ADCY1*. βy means *GNG7*.

We performed KEGG analysis on 13 selected genes in DAVID to explore the potential pathway and mechanism that 13 genes may regulate the occurrence and development of PAAD*. *The result showed 6 significant KEGG pathways for 13 validated genes, including Chemokine signaling pathway, Cytokine-cytokine receptor interaction, Rheumatoid arthritis, Pathways in cancer, Influenza A, and Herpes simplex infection (Table 1). We observed that two genes (*GNG7, ADCY1*) were meaningfully enriched in the Pathways in Cancer (*P* = 0.016, Fig. 5) (https://www.kegg.jp/kegg/kegg1.html).

**Table 1:** The KEGG enrichment pathways of 13 validated genes expression in DAVID database.

Category	Term	Count	*P value	Genes	Benjamini
KEGG_PATHWAY	hsa04062: Chemokine signaling pathway	4	< 0.001	*CCL20, GNG7, ADCY1, CXCL5*	< 0.001
KEGG_PATHWAY	hsa04060: Cytokine-cytokine receptor interaction	2	0.004	*CCL20, CXCL5*	0.135
KEGG_PATHWAY	hsa05323: Rheumatoid arthritis	2	0.007	*CCL20, CXCL5*	0.141
KEGG_PATHWAY	hsa05200: Pathways in cancer	2	0.016	*GNG7, ADCY1*	0.255
KEGG_PATHWAY	hsa05164: Influenza A	3	0.025	*RSAD2, OAS1, OAS2*	0.284
KEGG_PATHWAY	hsa05168: Herpes simplex infection	3	0.028	*C5, OAS1, OAS2*	0.284

*Diagnostic value and clinical outcomes of GNG7* and *ADCY1 in TCGA database.*

**Figure 6:**
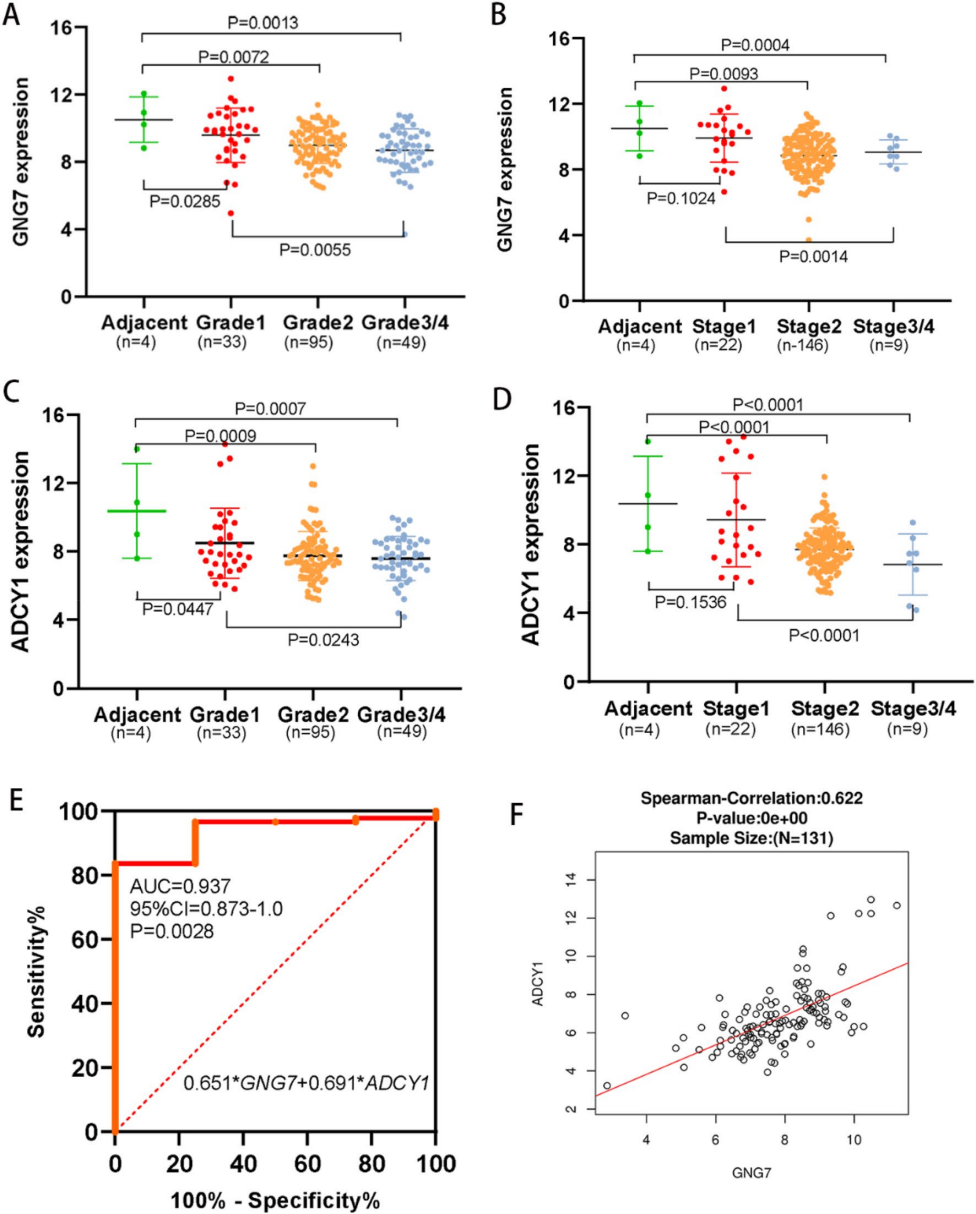
Analysis of *GNG7* AND *ADCY1* expression and diagnostic significance of 177 cases of PAAD patients in the TCGA database. (**A**, **B**) The mRNA expression of *GNG7* in the PAAD tissues was significantly lower than in the adjacent normal tissues. The *GNG7* mRNA expression was incrementally downregulated with increasing neoplasm histology grades (A) as well as tumor stages (**B**). (**C**–**D**) The mRNA expression of *ADCY1* in the PAAD tissues was significantly lower than in the adjacent normal tissues. The *ADCY1* mRNA expression was incrementally downregulated with increasing neoplasm histology grades (C) as well as tumor stages (**D**). (**E**) The ROC curve showed that the combined expression of *GNG7* and *ADCY1* has diagnostic value for PAAD patients (AUC = 0.937, *P* = 0.0028). (**F**) The Pearman correlation test analysis exhibited a significantly positive correlation between *GNG7* and *ADCY1* (r = 0.622, *P* = 0.000). This figure was drawn by GraphPad Prism 5.0 (GraphPad Software, Inc., San Diego, CA, USA; https://www.graphpad.com/).

We downloaded the PAAD data set from the TCGA database to analyze the relationship between *GNG7, ADCY1* mRNA expression and the clinical outcome. The mRNA expression of *GNG7* and *ADCY1* in the PAAD tissues was significantly lower than in the normal tissues. In addition, the *GNG7* and *ADCY1* mRNA expression was incrementally downregulated with increasing neoplasm histology grades as well as tumor stages (Fig. 6A–D). The low expression of *GNG7* mRNA was related to the neoplasm histology grades (*P* = 0.045) and survival (*P* = 0.007) in PAAD patients. The low expression of *ADCY1* mRNA was related to gender (*P* = 0.035), neoplasm histology grades (*P* = 0.008), and survival (*P* = 0.036) (Table 2). The Multivariate Cox Regression analysis showed that the *GNG7* (HR (95%CI): 2.106 (1.357–3.269); *P* = 0.010) and *ADCY1* (HR (95%CI): 1.881 (1.221–2.898); *P* = 0.004) low expression, neoplasm histology grades 3/4 (HR (95%CI): 0.637 (0.408–0.995); *P* = 0.047), preoperative pharmaceutical history (HR (95%CI): 0.452 (0.281–0.725); *P* = 0.001) were independent risk factors for overall survival of PAAD (Table 3). And we constructed a combined expression model of *GNG7* and *ADCY1* to evaluate the diagnostic value for PAAD. The Combined expression score = 0.651* *GNG7* + 0.691*ADCY1. The ROC analysis showed that combined expression of *GNG7* and *ADCY1* have diagnostic value in distinguishing between PAAD and normal pancreas tissue (AUC = 0.937, *P* = 0.0028, Fig. 6E). Finally, we performed the Pearman correlation test analysis in the LinkedOmics database^28^ to investigate the correlation of the mRNA expression between *GNG7* and *ADCY1* in the PAAD samples. The result exhibited a significantly positive correlation (r = 0.622, *P* = 0.000, Fig. 6F).

**Table 2:** Correlation between *GNG7, ADCY1* expression and clinical outcomes in PAAD (177cases, GDC TCGA pancreatic Cancer cohort).

Characteristics		*GNG7* level		X^2^	*P-Value	*ADCY1* level		X^2^	*P-Value
		High (n)	Low (n)			High (n)	Low (n)		
Gender	Male	44	53	2.270	0.132	42	55	4.450	0.035
	Female	45	35			47	33		
Age (years)	> 60	59	60	0.102	0.749	59	60	0.102	0.749
	< = 60	30	28			30	28		
Tumor stage	I/II	85	84	0.000	1.000	85	84	0.000	1.000
	III/IV	4	4			4	4		
Neoplasm histology grades	G1/G2	70	57	4.005	0.045	72	55	7.120	0.008
	G3/G4	19	31			17	33		
Family cancer history	Yes	41	46	0.809	0.368	46	41	0.360	0.549
	No	48	42			43	47		
Radiation	Yes	25	23	0.028	0.867	23	25	0.253	0.615
	No	64	65			66	63		
Pharmaceutical	Yes	64	63	0.000	1.000	62	65	0.445	0.505
	No	25	25			27	23		
Survival	Alive	52	33	7.289	0.007	51	36	4.405	0.036
	Dead	37	55			38	53

**Table 3:** Univariate and multivariate cox regression analysis of overall survival and recurrence-free survival (177 cases, GDC TCGA pancreatic cancer cohort).

Overall survival	Group	Univariate analysis	*P-Value	Multivariate analysis	*P Value
Variables		HR (95%CI)		HR (95%CI)	
*GNG7*	Low vs. high	1.959 (1.284–2.988)	0.003	2.106 (1.357–3.269)	0.010
*ADCY1*	Low vs. high	1.567 (1.037–2.369)	0.043	1.881 (1.221–2.898)	0.004
Tumor stage	I/II vs. III/IV	1.454 (0.438–4.611)	0.525	1.224 (0.382–3.296)	0.732
Neoplasm histology grades	G1/G2 vs. G3/G4	0.651 (0.422–1.002)	0.052	0.637 (0.408–0.995)	0.047
Radiation	Yes vs. no	0.542 (0.327–0.900)	0.018	0.624 (0.358–1.087)	0.096
Pharmaceutical	Yes vs. no	0.466 (0.304–0.714)	< 0.001	0.452 (0.281–0.725)	0.001

*Diagnostic and prognostic value of GNG7* and *ADCY1 was investigated in the* GSE84129 and GSE62452 datasets.

**Figure 7:**
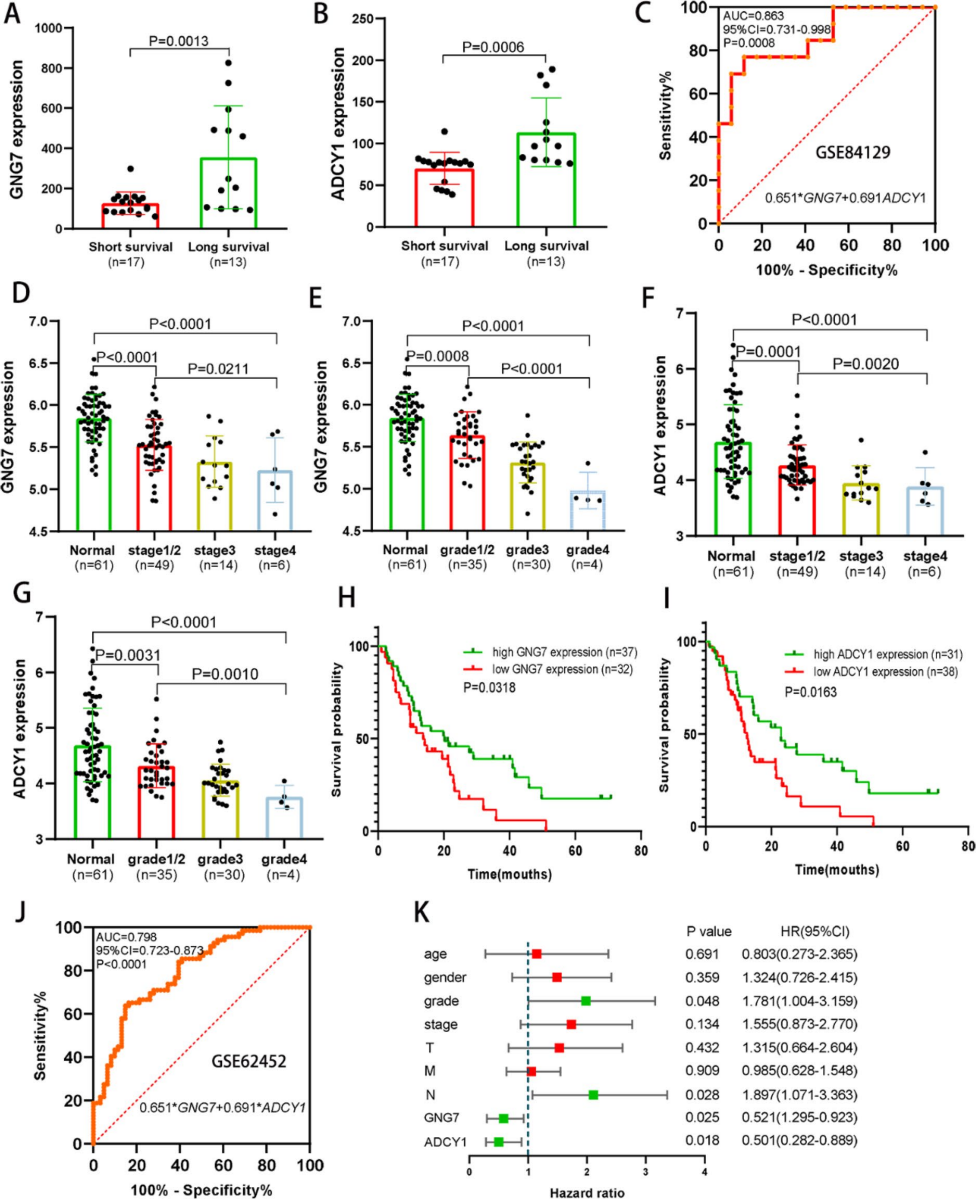
Diagnostic and prognostic value of GNG7 and ADCY1 in the GSE84129 and GSE62452 datasets. (**A**, **B**) PAAD patients with high survival time have significantly higher mRNA expression of *GNG7* (**A**) and *ADCY1* (**B**) than patients with short survival time. (**C**) The ROC curve showed that the combined expression of *GNG7* and *ADCY1* has diagnostic value for PAAD in the GSE84129 dataset (AUC = 0.863, *P* = 0.0008). (**D**–**E**) The *GNG7* (**D**) and *ADCY1* (**E**) mRNA expression was incrementally downregulated with increasing tumor stages. (**F**, **G**) The *GNG7* (**F**) and *ADCY1* (**G**) mRNA expression was incrementally downregulated with increasing neoplasm histology grades. (**H**, **I**) PAAD patients with high expression of *GNG7* (**H**) and *ADCY1* (I) have a longer survival time than patients with low expression. (**J**) The ROC curve showed that the combined expression of *GNG7* and *ADCY1* has diagnostic value for PAAD in the GSE62452 dataset (AUC = 0.798, *P* < 0.0001). (**K**) Low expression of GNG7 and ADCY1 were independent risk factors for OS of PAAD patients.

We downloaded two independent PAAD datasets from the GEO database (GSE84129 and GSE62452 datasets) to analyze the diagnostic and prognostic value of *GNG7* and *ADCY1*. In the GSE84129 dataset, PAAD patients with high survival time (more than 24 months) have significantly higher mRNA expression of *GNG7* and *ADCY1* than patients with short survival time (no more than 24 months) (Fig. 7A,B). In addition, the ROC analysis showed that combined expression of GNG7 and ADCY1 have diagnostic value in distinguishing between PAAD and normal pancreas tissue (AUC = 0.863, *P* = 0.0008, Fig. 7C). In the independent GSE62452 dataset, the *GNG7* and *ADCY1* mRNA expression was incrementally downregulated with increasing tumor stages as well as neoplasm histology grades (Fig. 7D–G). Furthermore, PAAD patients with high expression of *GNG7* and *ADCY1* have a longer survival time than patients with low *GNG7* and *ADCY1* expression (Fig. 7H,I). We also performed the ROC analysis showed that combined expression of *GNG7* and *ADCY1* have diagnostic value for HCC (AUC = 0.798, *P* < 0.0001, Fig. 7J). Moreover, the Multivariate Cox Regression analysis and the results showed that low expression of *GNG7* and *ADCY1* were independent risk factors for OS of PAAD patients (Fig. 7K). All these results demonstrated that the *GNG7* and *ADCY1* has diagnostic and prognostic value for PAAD.

## Discussion

PAAD is one of the most malignant cancers. Due to the lack of sensitive early diagnosis biomarker, it is challenging to develop effective treatments to delay and reverse the progression of PAAD. The 5-year survival percentage of PAAD patients for all stages was only 9%, based on data in 2019 released by the American Cancer Society^29^. Therefore, more meaningful biomarkers should be discovered to improve the diagnostic and prognostic efficiency of PAAD patients^30,31^. At present, a variety of biomarkers with prognosis and prognosis for PAAD has been reported^32–34^. Jiang et al. suggest that CDK1 and CCNA2 may be potential diagnostic and prognostic biomarkers in patients with PAAD^35^. In addition, CDK1 and CCNA2 has been reported promoting progression of PAAD via regulating the P53 and cell cycle signaling pathway, which were subpathways of Pathway in cancer^35–37^. Georgiadou et al. reported that overexpression of VEGF and Id-1 was associated with high microvessel density and emerged as prognostic factors in patient survival in PAAD^38^. Additionally, the VEGF signaling pathways take an important position in Pathway in cancer (Fig. 6).

This present study aimed to screen out the genes that has diagnostic and prognostic significance for PAAD by comparing the tumor tissues with adjacent non-malignant tissues. 118 up-regulated and 143 down-regulated overlapping DEGs were screened out from the microarray data GSE55643 and GSE15471. Then, we performed the GO and KEGG enrichment analysis for overlapping DEGs using the DAVID database. Subsequently, we constructed a PPI network with 186 nodes/genes and 348 edges in the STRING database and visualized it by Cytoscape software. From the PPI network, we screened out the two most core gene clusters, which including 13 up-regulated and 5 down-regulated genes. We analyzed the mRNA expression and the prognosis of survival of these genes in the TNMplot and Kaplan–Meier plotters database, respectively. By this process, 10 up-regulated and 3 down-regulated genes were screened. Next, we performed KEGG analysis on 13 selected genes in DAVID to explore the potential pathway and mechanism that 13 genes may regulate the occurrence and development of PAAD*.* We observed that two genes (*GNG7, ADCY1*) meaningfully enriched in the Pathways in Cancer. Additionally, we performed diagnostic and prognostic analysis of *GNG7* and *ADCY1* in the independent TCGA cohort, GSE84129 and GSE62452 datasets. The result showed that the mRNA expression of *GNG7* and *ADCY1* in the PAAD tissues was significantly lower than in the normal tissues. The Multivariate Cox Regression analysis showed that the low expression of *GNG7* (HR (95%CI): 2.106 (1.357–3.269); *P* = 0.010) and *ADCY1* (HR (95%CI): 1.881 (1.221–2.898); *P* = 0.004) were independent risk factors for overall survival of PAAD (Table 3). The ROC analysis showed that low combined expression of *GNG7* and *ADCY1* have diagnostic value in distinguishing between PAAD and normal pancreas tissue. Meanwhile, the Pearman correlation test analysis in the LinkedOmics database exhibited a significantly positive correlation between *GNG7* and *ADCY1* (r = 0.622, *P* = 0.000). We observed that the expression of GNG7 and ADCY1 in the TCGA database has no significant difference between adjacent normal tissue and tumors with early stages (stage 1) or tumor grades (grade 1). This lack of significance may relate to the small normal sample size (n = 4). After all, we notice a significantly different expression in the GSE62452 dataset between adjacent normal tissue and tumors with early stages or grades. However, the diagnostic efficiency of GNG7 and ADCY1 for early stage PAAD indeed requires further investigations. Therefore, all our results indicate that *GNG7* and *ADCY1* may as diagnostic and prognostic biomarker for PAAD.

*GNG7* are involved as a modulator or transducer in various transmembrane signaling systems. The beta and gamma chains are required for the GTPase activity, for replacement of GDP by GTP, and for G protein-effector interaction. Plays a role in the regulation of adenylyl cyclase signaling in certain regions of the brain^39^. A previous study showed that *GNG7* was down-regulated in clear cell renal cell carcinoma tissues due to promoter methylation and frequent gene mutations, and negatively associated with overall survival^40^. Additionally, previous studies showed that reduced expression of *GNG7* was associated with breast cancer, lung carcinogenesis, head and neck cancer and esophageal cancer^41–45^. However, rarely the previous study has addressed the role of *GNG7* in PAAD and its prognostic value. This study is the first systematic investigation of diagnostic value, clinical significance of *GNG7* in PAAD.

Adenylate cyclase 1 (*ADCY1*) encodes a member of the adenylate cyclase gene family that is primarily expressed in the brain^46,47^. This protein is regulated by calcium/calmodulin concentration and may be involved in brain development. *ADCY1* can regulate drug resistance in lung cancer through participating in cAMP signaling pathways and was of great significance to be a novel prognostic biomarker^48^. It is worth noting that the cAMP signaling pathways was the subpathways of Pathway in cancer. Additionally, the upregulation of *ADCY1* by regulating microRNA-127-3p exerts anti-tumor effects on colon cancer^49^. Y Li et al. showed that the expression of *ADCY1* was downregulated in osteosarcoma compared with benign bone tumors, suggesting that *ADCY1* may be potential biomarkers for osteosarcoma tumorigenesis and therapeutics^50^. Furthermore, previous studies reported that ADCY1 was a key candidate gene in melanoma and rectal adenocarcinoma metastasis^51,52^. However, Ma M et al. reported that ADCY1 was regulated by miR-23a-3p and plays a cancer‐promoting role in mucosal melanoma^53^.

In summary, this study screened out 2 potential genes that has diagnostic and prognostic significance for PAAD through the systematic bioinformatic-based analyses. Of course, all of the DEG candidate genes we have screened out related to PAAD should be confirmed through molecular biology and cytology experiments.

## Conclusion

The mRNA expression level of *GNG7, ADCY1* was significantly down-regulated in PAAD tissues compared with adjacent normal tissues. Low *GNG7, ADCY1* mRNA expression level was associated with poor clinical outcomes of PAAD patients. *GNG7* and *ADCY1* may be an oncogene that regulated tumorigenesis and development through pathways in cancer and could be used as a biomarker of the diagnostic and prognostic value of PAAD.

## Supplementary Information

Supplementary Legends.

Supplementary Figure 1.

Supplementary Tables.

## Data Availability

All datasets generated for this study are available within the article.
