# Supplementary material for: GNG7 and ADCY1 as diagnostic and prognostic biomarkers for pancreatic adenocarcinoma through bioinformatic-based analyses
Source: Sci Rep. 2021 Oct 14;11:20441. doi: 10.1038/s41598-021-99544-x (PMC8516928; doi:10.1038/s41598-021-99544-x)
Supplement: Supplementary file 1 — Supplementary Legends. [file 41598_2021_99544_MOESM1_ESM.docx]

Supplementary Figure. 1. GO terms and KEGG pathways of DEGs significantly enriched in PAAD. DEG, differentially expressed gene; GO, gene ontology; PAAD, pancreatic adenocarcinoma; KEGG, Kyoto Encyclopedia of Genes and Genomes.
