# Supplementary material for: GNG7 and ADCY1 as diagnostic and prognostic biomarkers for pancreatic adenocarcinoma through bioinformatic-based analyses
Source: Sci Rep. 2021 Oct 14;11:20441. doi: 10.1038/s41598-021-99544-x (PMC8516928; doi:10.1038/s41598-021-99544-x)
Supplement: Supplementary file 3 — Supplementary Tables. [file 41598_2021_99544_MOESM3_ESM.docx]

Supplementary table 1. 261 Differentially Expressed Genes Were Identified in GSE55643 and GSE15471, Including 118 Upregulated Genes and 143 Downregulated Genes in Samples of PAAD Patients

| DEGs | Gene name |
| --- | --- |
| Up-regulated genes | *COL6A3, COL11A1, ANO1, SLPI, ITGA2, TGM2, KRT7, ANXA2, GPRC5A, LAMC2, AHNAK2, GJB2, LRRN1, MBOAT2, PHLDA2, DCBLD2, ASAP2, DGKH, CKLF, MMP11, CXCL5, RSAD2, EFNA5, S100P, SLC9B2, INPP4B, FXYD5, TRIM29, IL1RAP, DKK1,PCDH7, HK2, XAF1, KLK10, BST2, SFN, HS3ST1, ARNTL2, SLC6A14, IFI27, SYTL2, TRIM59, LAMA3, CDH3, IL1RN, ITGA3, LCN2, WFDC2, ADAMTS6, TMPRSS3, NQO1, IFIT2, LRRC15, RAPH1, SFTA2, CTSE, TFAP2A, ULBP2, MMP28, IGF2BP3, SDR16C5, FERMT1, SLC2A1PIEZO2, CXCL3, KCNN4, TSPAN1, NMU, OAS2, RHBDL2, MSLN, SERPINB5, FOXQ1, TMPRSS4, MLPH, C19orf33, FXYD3, CAPN8, ANLN, CENPF, IFI6, HOTAIRM1, CLDN18, CCL20, ASPHD2, TRNP1, SCEL, GPR110, S100A2, LEMD1, SOX11,STYK1, GALNT5, ASPM, HMGA2, VSIG1, GBP3, FAM83D, CXCL10, CENPK, NEK2, ANXA10, MUC5B, TFF1, DPCR1, DLGAP5, EREG, S100A14, GPR87, ZG16B, PLAC8, GJB6, SULT1C2, KRT6A, OAS1, HRH1, ITGB8, PFKFB3* |
| Down-regulated genes | *BTD, SPINK2, GSTA3, CCDC69, EPHA8, FAM46C, PGM5, AMHR2, TBC1D21, DNASE1, C1orf194, ATOH1, MYRIP, RHD, MRO, IRF4,RET, LRFN1, GNG7, PACSIN1, CBFA2T3, KLK12, BRSK2, ZG16, CACNA1I, EPB41L4B, MCOLN3, CELF4, VIPR2, LPAR3, MPV17L,NGB, BPIFA3, ESR1, ECE2, PAIP2B, RAB39B, WFDC8, C5, KLB, PSAT1, DPP10, ARHGDIG, PDK4, DMD, ADCY1, ECHDC3, KSR1, GDF10, GUCA1C, CNTD1, GPR123, ABCC13, CA4, MOGAT2, LIFR, SLC8A3, ZNF503, AS1, WNK2, MTUS2, ATP4A, GTSF1L, FLT3,COCH, BTG2, MYOC, BTNL3, KIAA1324, RNF186, CCDC110, SLC8A2, MLXIPL, CHN2, SLC1A2, CIDEA, CLDND2, KCNK3, GNMT,KIAA1958, SLC35G1, TEX11, CTNND2, FLJ38379, RNF212, GLS2, LOC1, G6PC, SLC30A2, PLA2G12B, SLC17A4, SYT6, ERO1LB,BCAT2, UNC79, MGAT4A, BNIP3, RADIL, ENPP3, NRCAM, GUCA2A, DPEP1, LOC100, SERPINI1, SERP1, LINC00339, DNASE1L3,TMED6, DDC, NR5A2, ACADL, FAM107A, TTYH1, GSTA1, STXBP6, ART3, TMPRSS15, ANGPTL1, AOX1, MEP1A, CLDN3, XPNPEP2,SULT2A1, FAM150B, FGL1, SYBU, SERPINI2, ERP27, ISX, APOA1, PNLIPRP1, CD160, MEP1B, CXCL12, S100G, TSPAN7, PLIN1, HS6ST2, ZBTB16, RBP2, SLC26A3, FLJ22763, MTTP, DPT* |

Supplementary table 2. The Top three Pathways in GO and KEGG Enrichment Analysis of DEGs

| Category | Term | Count | **P*-Value |
| --- | --- | --- | --- |
| Upregulated DEGs |  |  |  |
| GOTERM_BP_DIRECT | GO:0060337~type I interferon signaling pathway | 8 | 2.08E-07 |
| GOTERM_BP_DIRECT | GO:0009615~response to virus | 7 | 8.93E-05 |
| GOTERM_BP_DIRECT | GO:0030198~extracellular matrix organization | 8 | 3.24E-04 |
| GOTERM_CC_DIRECT | GO:0005615~extracellular space | 21 | 2.15E-04 |
| GOTERM_CC_DIRECT | GO:0070062~extracellular exosome | 31 | 1.58E-03 |
| GOTERM_CC_DIRECT | GO:0005576~extracellular region | 20 | 4.64E-03 |
| GOTERM_MF_DIRECT | GO:0008009~chemokine activity | 5 | 2.50E-04 |
| GOTERM_MF_DIRECT | GO:0043236~laminin binding | 3 | 1.07E-02 |
| GOTERM_MF_DIRECT | GO:0005509~calcium ion binding | 11 | 1.47E-02 |
| KEGG_PATHWAY | hsa04512: ECM-receptor interaction | 7 | 9.35E-06 |
| KEGG_PATHWAY | hsa04510: Focal adhesion | 7 | 1.10E-03 |
| KEGG_PATHWAY | hsa04151: PI3K-Akt signaling pathway | 8 | 3.34E-03 |
| Downregulated DEGs |  |  |  |
| GOTERM_BP_DIRECT | GO:0072593~reactive oxygen species metabolic process | 5 | 1.13E-04 |
| GOTERM_BP_DIRECT | GO:0030155~regulation of cell adhesion | 4 | 0.0036507 |
| GOTERM_BP_DIRECT | GO:0010259~multicellular organism aging | 3 | 0.0065252 |
| GOTERM_CC_DIRECT | GO:0005887~integral component of plasma membrane | 24 | 1.40E-04 |
| GOTERM_CC_DIRECT | GO:0070062~extracellular exosome | 34 | 0.0018665 |
| GOTERM_CC_DIRECT | GO:0016021~integral component of membrane | 50 | 0.0109534 |
| GOTERM_MF_DIRECT | GO:0008289~lipid binding | 5 | 0.0233561 |
| GOTERM_MF_DIRECT | GO:0005543~phospholipid binding | 4 | 0.0239005 |
| GOTERM_MF_DIRECT | GO:0004867~serine-type endopeptidase inhibitor activity | 4 | 0.0325524 |
| KEGG_PATHWAY | hsa04975: Fat digestion and absorption | 5 | 3.00E-04 |
| KEGG_PATHWAY | hsa04974: Protein digestion and absorption | 5 | 0.0062716 |
| KEGG_PATHWAY | hsa04977: Vitamin digestion and absorption | 3 | 0.0144846 |

GO, gene ontology; KEGG, Kyoto Encyclopedia of Genes and Genomes.
